# Saint-Bonnet Doppler waveform classification system: a scoping review and evidence-based appraisal using AGREE II and AGREE-REX

**DOI:** 10.3389/fcvm.2026.1870182

**Published:** 2026-10-14

**Authors:** Hamidah Mabruk Alodayni, Fatimah Alodaini, Alun H. Davies, Pasha Normahani

**Affiliations:** 1 Section of Vascular Surgery, Department of Surgery and Cancer, Imperial College London, London, United Kingdom; 2 Department of Radiology and Medical Imaging, College of Applied Medical Sciences, Majmaah University, Al-Majmaah, Saudi Arabia; 3 Institute of Epidemiology and Health Care, University College London (UCL), London, United Kingdom; 4 Imperial Vascular Unit, Imperial College Healthcare NHS Trust, London, United Kingdom

**Keywords:** AGREE II, AGREE-REX, Doppler waveform, duplex ultrasound, hand-held Doppler, peripheral arterial disease, Saint-Bonnet, scoping review

## Abstract

**Background:**

Doppler waveform interpretation is central to the diagnosis and surveillance of lower limb peripheral arterial disease (PAD). Despite widespread clinical use of duplex ultrasound (DUS) and hand-held Doppler (HHD), waveform terminology remains highly variable and non-standardised across institutions. The Saint-Bonnet classification, proposed by the French College of Teachers in Vascular Medicine (CEMV), offers a structured eight-category morphological framework mapped to haemodynamic severity. Its evidence base and methodological quality have never been formally appraised.

**Methods:**

A scoping review was conducted following the Preferred Reporting Items for Systematic Reviews and Meta-Analyses extension for Scoping Reviews (PRISMA-ScR). MEDLINE (Ovid) and Embase (Ovid) were searched from 1946 to November 2025. Studies describing, applying, validating, or evaluating the Saint-Bonnet classification were eligible. Data were extracted using a standardised form. Methodological quality was appraised using AGREE II (six domains) and AGREE-REX (three domains), independently by two reviewers (H.A. and F.A.).

**Results:**

Seven studies met the inclusion criteria; all published between 2017 and 2022 from a single French vascular research group. The Saint-Bonnet classification achieved a categorisation rate of 98.2% across 1,033 waveforms, outperforming alternative systems. Inter-rater reliability was moderate (*κ* ≈ 0.55). Waveform grade independently correlated with maximal walking distance, and waveform-guided ankle–brachial index (ABI) selection achieved near-perfect agreement with guideline standards (*κ* up to 1.00). Automated neural network classification reached *κ* = 0.79, approaching expert agreement. AGREE II appraisal revealed high scores for Clarity of Presentation and Scope & Purpose (97% and 83%), but low scores for Rigour of Development and Applicability (44% and 40%). AGREE-REX demonstrated high Clinical Applicability and Implementability.

**Conclusion:**

The Saint-Bonnet classification is a promising and practical framework for Doppler waveform interpretation, with consistent findings across the available studies. Low AGREE II scores for Rigour of Development and Applicability reflect gaps in its documented development and reporting rather than evidence against its clinical performance. However, all supporting evidence originates from a single, non-independent French research group, and the classification has not undergone external validation; broader adoption should await independent, multicentre confirmation of these findings.

## 1 Introduction

Lower limb peripheral arterial disease (PAD) is a common manifestation of systemic atherosclerosis, affecting more than 200 million people worldwide (1, 2). Early diagnosis and surveillance after revascularisation procedures are essential to prevent complications such as limb loss (3). Duplex ultrasound (DUS) is the most widely used primary imaging modality for PAD diagnosis and post-revascularisation surveillance, offering detailed anatomical and haemodynamic information through B-mode imaging and Doppler waveform analysis (4). Whether obtained by DUS or hand-held Doppler (HHD), Doppler waveform assessment provides real-time arterial haemodynamic information by analysing waveform morphology and remains a cornerstone of vascular assessment in both clinical and post-revascularisation settings (4). Despite its widespread use, Doppler waveform interpretation is highly operator-dependent and inconsistent across institutions (5, 6). There are multiple descriptive terms commonly employed in practice (such as triphasic, biphasic, monophasic), but there is no standardised classification system that is universally accepted (7–9). These differences affect reproducibility, reduce diagnostic accuracy and contribute to inter-observer disagreements (6, 10, 11). To bring some level of consistency, a proposal was made by Collège des Enseignants de Médecine Vasculaire (CEMV), which is the French College of Teachers for Vascular Medicine, whereby a structured Doppler waveform classification was introduced called the Saint-Bonnet. This is classified into eight morphological types (N, A, B, CD, E, O, FA, and U) beginning from normal triphasic waveforms to absence of flow, including unusual forms (12). Although frequently cited in the field of vascular medicine, very little information exists on the use and methodological quality of this classification method, and no appraisal of its validity and reproducibility across various clinical pathways has yet been performed.

A systematic map of evidence relating to the development, use, validity, and methodology of the Saint-Bonnet waveform classification has not been conducted before, and more importantly, the quality of this waveform classification has not been appraised using standard guidelines for recommendations such as AGREE II (Appraisal of Guidelines for Research and Evaluation II) and AGREE-REX (Recommendation EXcellence).

This scoping review therefore aims to: (1) identify and summarise all studies that describe, apply, validate, or evaluate the Saint-Bonnet Doppler waveform classification system; and (2) assess the methodological quality and clinical applicability of the classification using both AGREE II and AGREE-REX appraisal tools. By synthesising existing evidence and evaluating the quality of the framework, this review provides clarity on whether the Saint-Bonnet classification is suitable for standardising Doppler waveform interpretation in clinical and research practice.

## 2 Methods

### 2.1 Study design

A scoping review was conducted following the Preferred Reporting Items for Systematic Reviews and Meta-Analyses extension for Scoping Reviews (PRISMA-ScR) (14). Scoping reviews are particularly appropriate when the aim is to map the extent, range, and nature of evidence on a topic, especially where the evidence base is heterogeneous and a formal systematic review or meta-analysis is not feasible.

### 2.2 Research question and eligibility criteria (PICOS)

Eligibility criteria were defined using the PICOS framework. (P) Population: patients with lower limb peripheral arterial disease (PAD) undergoing clinical or post-revascularisation Doppler assessment in any healthcare setting. (I) Index classification: the Saint-Bonnet arterial Doppler waveform classification system, in either its original 13-category or simplified 8-category form. (C) Comparators: alternative Doppler waveform classification systems (e.g., Descotes, Spronk, Cathignol), expert free-text descriptions, or absence of a structured classification framework. (O) Outcomes: categorisation coverage rate, inter-rater reliability, diagnostic accuracy, functional or clinical associations, implementation and training experience, and automated or machine-learning performance. (S) Study designs: all study designs were eligible, including guideline and expert consensus documents, observational studies, prospective cohort studies, and computational proof-of-concept analyses, without language or date restrictions. Reporting followed the PRISMA extension for Scoping Reviews (PRISMA-ScR) (14).

### 2.3 Search strategy

This scoping review was not prospectively registered. A systematic search was performed in MEDLINE (Ovid) and Embase (Ovid) from 1946 to November 2025. The search strategy incorporated controlled vocabulary and free-text terms relating to Doppler ultrasound, waveform morphology, and classification terminology, including “Saint-Bonnet”, “Doppler waveform classification”, “hand-held Doppler”, and “continuous-wave Doppler”. No language restrictions, date limits, or study design filters were applied. The full search strategy is presented in Supplementary Table S1.

### 2.4 Eligibility criteria

Studies were eligible if they:

- described, applied, or evaluated the Saint-Bonnet arterial Doppler waveform classification;
- used the classification in clinical practice, education, or training;
- assessed inter-observer reproducibility, diagnostic accuracy, or implementation; or
- used the classification as a reference framework (e.g., for automated waveform interpretation or machine learning).

Exclusion criteria included: animal studies; non-arterial Doppler assessments; conference abstracts without accessible full text; and articles not referring to the Saint-Bonnet classification.

### 2.5 Study selection

Search results were imported into Covidence and deduplicated. Two reviewers (H.A. and F.A.) independently screened titles and abstracts, followed by full-text review of potentially eligible studies. Disagreements were resolved by discussion and consensus.

### 2.6 Data extraction

Data extraction was performed independently by two reviewers (H.A. and F.A.), with discrepancies resolved by discussion and consensus, consistent with the study selection process described in Section 2.5. Data were extracted using a standardised Microsoft Excel form. Extracted fields included:

- study characteristics (author, year, design, setting);
- how the Saint-Bonnet classification was applied (full or simplified version);
- waveform measurement protocol;
- diagnostic or clinical application; and
- any evaluation of reliability, diagnostic accuracy, or automation.

### 2.7 Quality appraisal: AGREE II and AGREE-REX

While quality assessment is not required for scoping review studies, the Saint-Bonnet classification system was appraised using two guideline appraisal instruments as follows:

AGREE II (Appraisal of Guidelines, Research and Evaluation II): an instrument that examines the methodology of guidelines in six dimensions (Scope and Purpose, Stakeholder Involvement, Rigour of Development, Clarity of Presentation, Applicability, and Independence). The two raters (H.A. and F.A.) scored the items independently on a 7-point Likert scale. Each dimension's score was converted into a percentage based on the AGREE II equation (15).

AGREE-REX (Recommendation EXcellence): measures the Clinical Applicability, Implementability, and Alignment with Values and Preferences (16).

The document appraised was the CEMV national recommendation (12), representing the formal guideline-format publication of the classification; the 2017 statement (17) was treated as its foundational document and considered alongside it during appraisal. Although AGREE II and AGREE-REX were developed primarily for appraising clinical practice guidelines, they were considered appropriate here because the Saint-Bonnet classification was formally issued as a CEMV national recommendation, meeting the structural criteria that these tools are designed to evaluate. No modifications were made to the standard AGREE II or AGREE-REX item sets or scoring procedures.

Both AGREE II and AGREE-REX explicitly state that no empirical data currently link specific quality scores to implementation or clinical outcomes, and that threshold-setting is therefore left to the discretion of appraisal teams (15, 16). Both manuals recommend that any such thresholds be defined *a priori*, by consensus among appraisers, before appraisal begins. Following this guidance, the two reviewers defined categorical interpretations (high/moderate/low quality) in advance for both instruments: for AGREE II, the ≥80%/60%–79%/<60% convention commonly adopted in guideline appraisal literature; and for AGREE-REX, an analogous three-tier threshold (≥5.0/3.5–4.9/<3.5 on the 7-point domain-mean scale), consistent with the consensus-based approach the AGREE-REX manual itself illustrates.

Inter-rater agreement between the two reviewers was assessed using the intraclass correlation coefficient (ICC, two-way random-effects, absolute agreement). Item-level scores for both reviewers are reported in Supplementary Table S2.

### 2.8 Data synthesis

As there was heterogeneity between studies regarding design and outcome measures, descriptive analysis was used for the synthesis of results. Meta-analysis was not conducted. The initial classification of 13 categories from Saint-Bonnet et al. has been reduced to a clinical classification of eight categories. A detailed category mapping is provided in Supplementary Table S3.

## 3 Results

### 3.1 Study selection

The database search yielded 16 records. After removal of seven duplicates, nine unique records underwent title and abstract screening. Seven full texts were retrieved and assessed for eligibility; all met the inclusion criteria and were included in the review. The selection process is illustrated in Figure 1.

**Figure 1:**
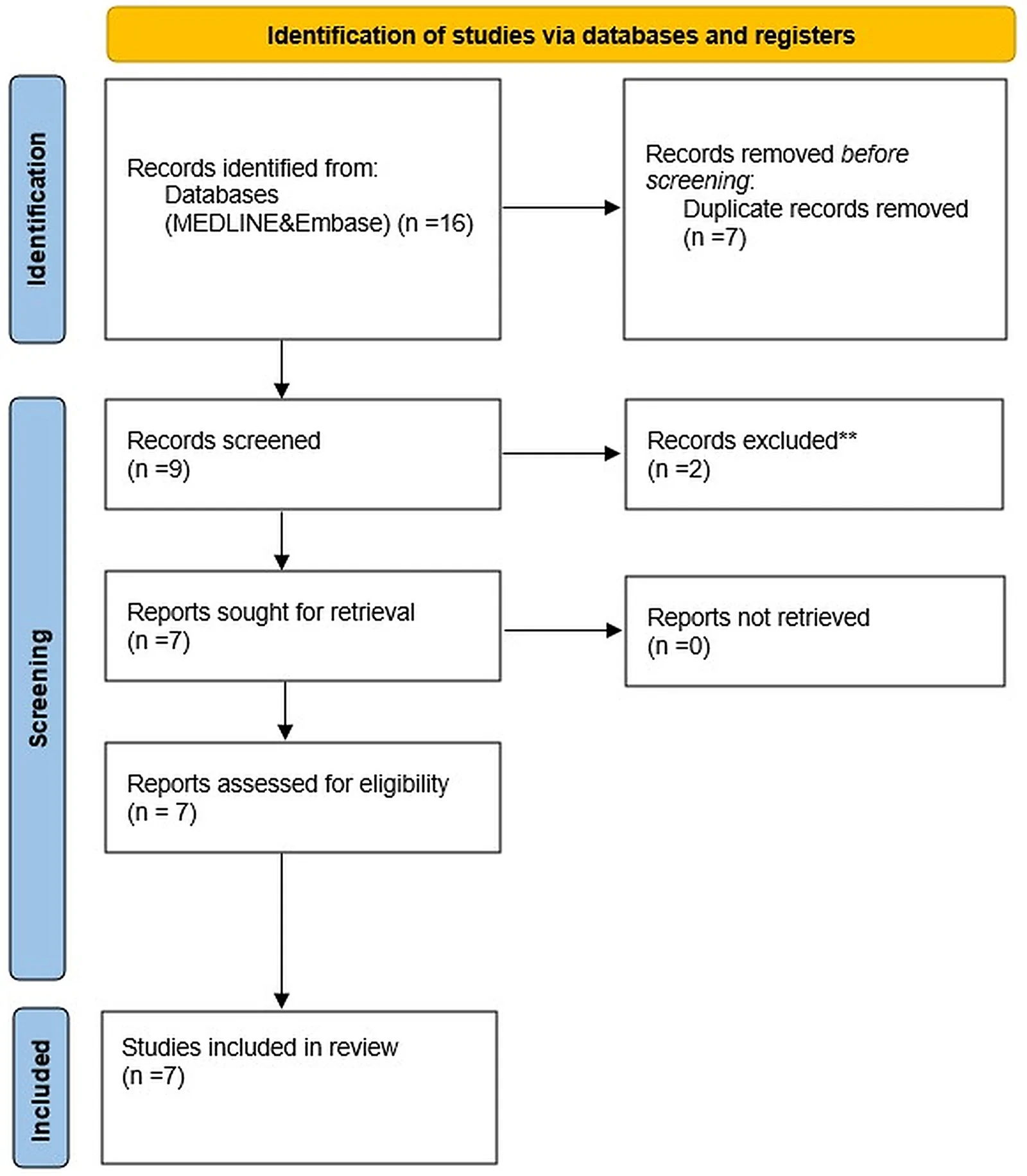
PRISMA-ScR flow diagram of the study selection process. Adapted from Page et al. (21), CC BY 4.0.

### 3.2 Characteristics of included studies

The seven included studies were published between 2017 and 2022, all originating from the same vascular research group in France. Two publications introduced and refined the Saint-Bonnet system (12, 17). One national observational study assessed inter-rater reliability among vascular physicians (11). One multicentre prospective study compared the classification against other waveform systems (5). One cross-sectional study examined association with functional outcomes (18). A prospective cohort investigated waveform morphology alongside ABI (19). A machine-learning proof-of-concept used the classification as reference labels for automated categorisation (20). Characteristics and key findings are summarised in Table 1.

**Table 1:** Characteristics and key findings of included studies.

Study (year)	Design	Objective	Application of Saint-Bonnet	Key findings
Mahé et al. (2017) (17)	Guideline/Expert Statement	Describe and justify a unified Doppler waveform classification	Introduced simplified Saint-Bonnet system based on haemodynamic severity	Identified major terminology heterogeneity; proposed structured 8-type classification
Mahé et al. (2018) (12)	National Guideline Update (CEMV)	Standardise Doppler waveform teaching and reporting	Recommended simplified Saint-Bonnet as national educational standard	Formal endorsement for routine clinical and training use in France
Lanéelle et al. (2021) (11)	National Observational Study	Assess descriptive variability and inter-rater reliability	110 clinicians classified 20 stored waveforms with and without Saint-Bonnet	Descriptive variability reduced from ∼93 to ∼6 expressions; inter-rater reliability moderate (*κ* ≈ 0.55)
Guilcher et al. (2021) (5)	Multicentre Prospective Study	Compare classification coverage across systems	All 1,033 waveforms classified using Descotes, Spronk, and Saint-Bonnet	Saint-Bonnet achieved highest categorisation rate (98.2%), outperforming existing systems
Miossec et al. (2021) (18)	Cross-Sectional Clinical Study	Examine association between waveform class and functional limitation	Simplified Saint-Bonnet used to grade resting Doppler waveforms in 186 patients	Waveform grade independently associated with maximal walking distance (*p* < 0.01)
Métairie et al. (2022) (19)	Prospective Cohort Study	Evaluate simplified ABI method guided by waveform morphology	Saint-Bonnet used to select best-flow artery for ABI in 188 patients	ABI using Saint-Bonnet achieved near-perfect agreement with guideline ABI (κ up to 1.00)
Guilcher et al. (2021) (20)	Machine-Learning Proof-of-Concept	Train and test automated classifier using Saint-Bonnet labelled images	Saint-Bonnet categories used as ground-truth labels for neural network training	Automated accuracy 83.7%; *κ* = 0.79, approaching expert agreement (*κ* = 0.83)

### 3.3 Results of individual sources of evidence

#### 3.3.1 Conceptual development

Two foundational papers established and formalised the Saint-Bonnet classification (12, 17). Mahé et al. highlighted major heterogeneity in Doppler waveform terminology and proposed the simplified Saint-Bonnet system comprising eight ordered waveform types reflecting haemodynamic severity. Mahé et al. formalised this through national CEMV recommendations, establishing it as the standard framework for vascular medicine trainees in France.

#### 3.3.2 Usability and inter-observer reliability

In a multicentre prospective study of 1,033 Doppler waveforms, Guilcher et al. (2021) found that the simplified Saint-Bonnet classification achieved a categorisation rate of 98.2%, significantly exceeding those of Spronk (91.3%) and Descotes & Cathignol (76.8%) classifications (*p* < 0.0001) (5).

Lanéelle et al. evaluated waveform interpretation practices among 110 vascular physicians using 20 anonymised waveforms, comparing free-text descriptions with the 13-category Saint-Bonnet system. Without the framework, clinicians produced a mean of 92.8 distinct descriptive expressions; this was reduced to 6.1 with Saint-Bonnet. Overall inter-rater reliability was moderate (*κ* = 0.546; 95% CI 0.544–0.547; *p* < 0.0001), with individual category agreement ranging from weak to strong (*κ* 0.24–0.87) (11).

#### 3.3.3 Functional association and ABI measurement applications

Miossec et al. demonstrated that Saint-Bonnet waveform grade was independently associated with maximal walking distance in 186 symptomatic PAD patients (*p* = 0.0009), outperforming ABI in multivariable models (18).

Métairie et al. applied Saint-Bonnet grading to streamline ABI measurement in 188 patients. Selecting only the ankle artery with the best-graded waveform produced ABI values with near-perfect agreement with guideline-standard ABI: *κ* = 0.95 (right limb) and 0.91 (left limb), with only 2.7%–4.3% discordant classifications, rising to *κ* = 1.00 when the two ankle arteries showed different waveform grades (19).

#### 3.3.4 Automated and machine-learning applications

Guilcher et al. trained a deep convolutional neural network using Saint-Bonnet-labelled waveform images. The automated system achieved an accuracy of 83.7% and *κ* = 0.79, approaching human expert consensus (*κ* = 0.83) (20).

### 3.4 Assessment of the Saint-Bonnet framework using AGREE II and AGREE-REX

Methodological quality was assessed using two validated appraisal tools applied to the CEMV guideline documentation. Given that the included studies comprised guideline documents, observational studies, and proof-of-concept analyses, conventional risk-of-bias instruments (e.g., RoB 2, ROBINS-I) were not applicable to the individual studies and were not used; the AGREE II and AGREE-REX tools were applied specifically to appraise the guideline/classification document itself, not as a risk-of-bias assessment of the included studies' internal validity. Results are summarised in Table 2 and illustrated in Figures 2A,B.

**Table 2:** AGREE II and AGREE-REX appraisal results for the Saint-Bonnet waveform classification system.

AGREE II		
Domain	Scaled domain score (%)	Interpretation
Domain 1: Scope and Purpose	83%	High quality
Domain 2: Stakeholder Involvement	69%	Moderate quality
Domain 3: Rigour of Development	44%	Low quality
Domain 4: Clarity of Presentation	97%	High quality
Domain 5: Applicability	40%	Low quality
Domain 6: Editorial Independence	50%	Low quality
AGREE-REX		
Domain	Mean domain score	Interpretation
Domain 1: Clinical Applicability	6.0	High quality
Domain 2: Values & Preferences	4.3	Moderate quality
Domain 3: Implementability	5.5	High quality

Author-defined interpretive thresholds, established a priori as recommended by the AGREE II User's Manual (15): scores ≥ 80% = high quality; 60–79% = moderate quality; <60% = low quality. Author-defined interpretive thresholds, established a priori by consensus as illustrated in the AGREE-REX manual (16): scores ≥ 5.0 = high quality; 3.5–4.9 = moderate quality; <3.5 = low quality.

**Figure 2:**
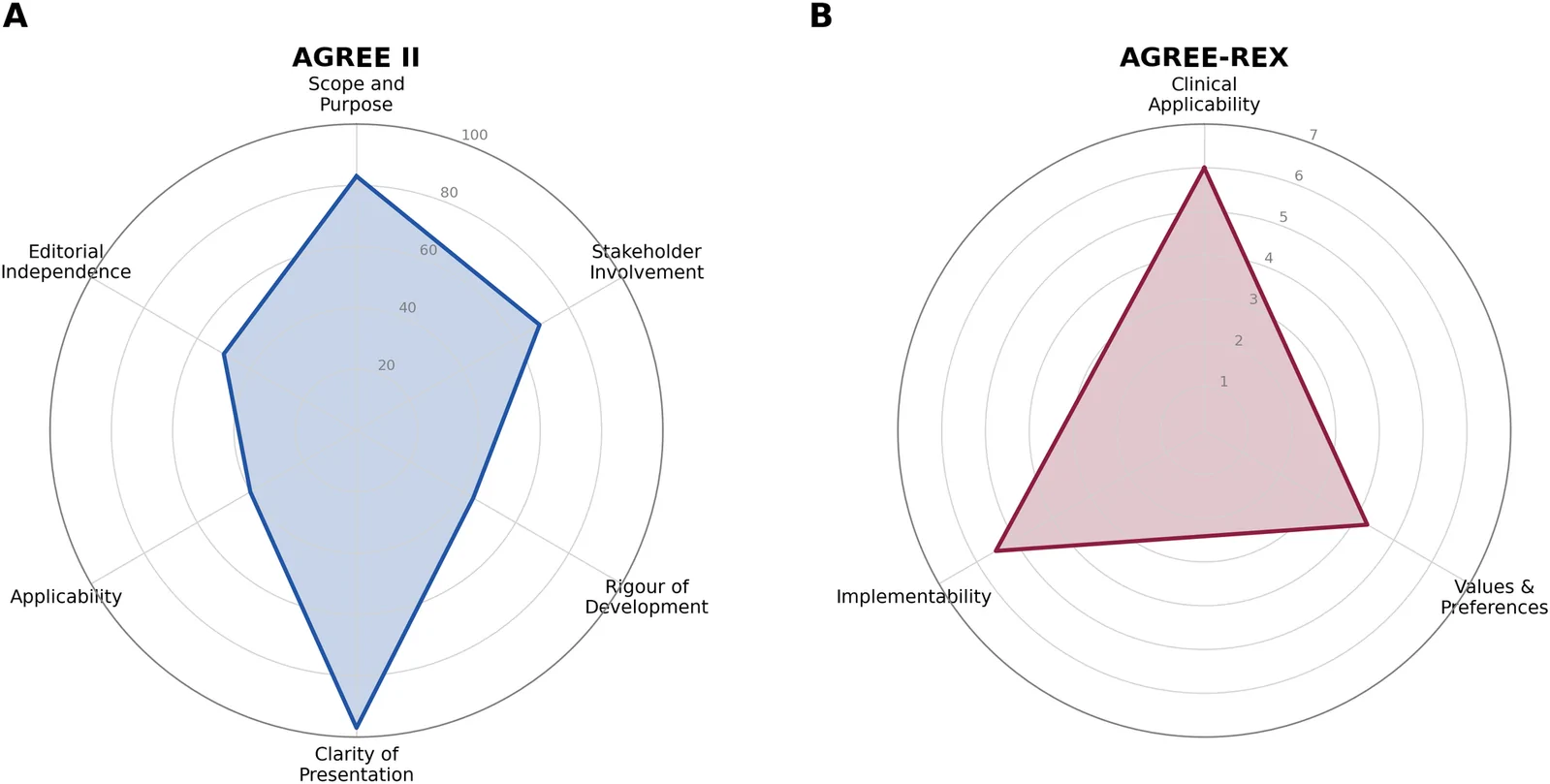
**(A)** Radar-chart visualisation of AGREE II domain scores applied to the Saint-Bonnet classification system. **(B)** Radar-chart visualisation of AGREE-REX domain scores applied to the Saint-Bonnet classification system.

AGREE II appraisal identified variability in methodological quality. Clarity of Presentation and Scope & Purpose received high scores (97% and 83%, respectively), reflecting well-defined aims and clear visual presentation of the classification stages. In contrast, Applicability and Rigour of Development received low scores (40% and 44%), indicating limited implementation planning, absence of systematic evidence synthesis, and minimal conflict-of-interest reporting. Stakeholder Involvement and Editorial Independence were rated at 69% and 50%, respectively.

AGREE-REX appraisal demonstrated high scores for Clinical Applicability (mean 6.0) and Implementability (5.5). Values & Preferences received a moderate score (4.3).

## 4 Discussion

This scoping review synthesised evidence on the development, application, and methodological quality of the Saint-Bonnet arterial Doppler waveform classification system. Across seven studies spanning conceptual development, clinical validation, and machine-learning applications the classification demonstrated clinical practicality and growing technological relevance. However, methodological robustness and generalisability remain limited.

### 4.1 Interpretation of findings

The Saint-Bonnet classification emerged in response to long-standing heterogeneity in Doppler waveform terminology. The initial papers (12, 17) pointed out the problems in description and suggested a systematic approach based on morphology correlated to haemodynamic severity. The national support provided by CEMV in France indicates early adoption of this approach in the clinic. The initial 13-class classification system was then refined to eight classes to make it more user-friendly; most of the reviewed papers utilised this system, except Lanéelle et al. (11).

The effectiveness of clinical assessments also reinforces the validity of the system. According to Guilcher et al., the categorisation rate reached 98.2%, far above other systems like Spronk and Descotes & Cathignol (5). Similarly, Lanéelle et al. found that unstructured analysis generated nearly 93 distinct statements, which were then distilled to only six through structured application of Saint-Bonnet (11). While inter-rater agreement was only moderate (*κ* ≈ 0.55), variation across stages suggested dependency on learning due to the system's recent development.

The classification also demonstrated meaningful functional and measurement associations. Miossec et al. established that waveform grades were associated with maximal walking distance independently, suggesting a role for waveform grading in functional assessment, independent of ABI categorisation (18). As this evidence derives from a cross-sectional design, it demonstrates an association with functional limitation rather than a confirmed prognostic role, which would require longitudinal follow-up. Furthermore, Métairie et al. demonstrated near-perfect agreement between waveform-guided ABI selection and guideline-standard ABI measurement (*κ* up to 1.00) (19). In relation to the automated waveform classification, Guilcher et al. found that a deep convolutional neural network trained on Saint-Bonnet-labelled data was able to perform at a level comparable to that of humans (*κ* = 0.79 compared to 0.83) (20). It should be noted that this result demonstrates the technical reproducibility of Saint-Bonnet categorisation by an automated model trained on expert-assigned labels, rather than independent clinical validation of the classification's diagnostic accuracy, since the reference standard used for training was the classification itself rather than an external clinical outcome.

### 4.2 Methodological strengths, weaknesses, and implications

AGREE II assessment showed inconsistency in terms of methodological quality. Areas such as scope, purpose, and clarity were highly rated since there was solid conceptual grounding. On the other hand, poor ratings for Rigour of Development, Applicability, and Editorial Independence signalled an absence of systematic evidence regarding the definition of categories, inadequate implementation plans, and conflict of interest disclosures. Notably, none of the sources mentioned patients' involvement in the process or educational design.

On the contrary, AGREE-REX assessment indicated high ratings for Clinical Applicability and Implementability, suggesting that clinicians find the system useful and clinically meaningful. Such a discrepancy, where low rigour in the developmental stage goes together with high clinical utility, is common for expert-driven systems promoted without adequate validation first.

### 4.3 Comparison with existing literature

The useability problems associated with other Doppler classification systems, such as the Descotes, Spronk, and Cathignol classification models, has also been noted by researchers. There have been numerous studies demonstrating that the Saint-Bonnet classification system achieves a higher categorisation rate than other alternative classifications (5). Despite the increasing awareness of the role of waveform morphology in PAD as reflected by international guidelines (13), a consistent Doppler classification system is yet to be adopted. The present review indicates that the Saint-Bonnet classification system may be a suitable candidate.

### 4.4 Gaps and research priorities

- The evidence base is limited in source, scope, and methodological approach. All seven articles were written by the same group of specialists from France, which reduces generalisability. Just one reliability study assessed independent clinician rating; there have been no external validation studies. Implementation studies have not investigated issues related to training costs, efficiency, and patient impact. Areas for further investigation should focus on:
- multicentre validation with international vascular sonographers and clinicians;
- head-to-head comparison across different healthcare systems and cultural contexts;
- longitudinal prognostic studies assessing waveform grade as a predictor of clinical outcomes; and
- integration into automated AI-assisted Doppler assessment platforms.

Structured educational materials must also be developed because of the moderately reliable findings seen during its initial application. The shortcomings of AGREE II emphasise the importance of systematic evidence integration, stakeholder involvement (patients included), and good governance prior to endorsing guidelines.

### 4.5 Strengths and limitations of this review

One of the strengths is the methodical identification of all available evidence from published sources by performing a double methodology assessment through AGREE II and AGREE-REX, which is one of the first reviews applying these two tools to Doppler waveforms classification. A key limitation of this review is that all seven included studies originated from a single French vascular research group, meaning the evidence base while internally consistent has not been independently replicated or geographically diversified. This constrains the generalisability of our findings and is a central reason multicentre, international validation is identified as a priority in Section 4.4. The weaknesses also stem from the limited number of studies involved in the analysis, which were mainly observational in design. No grey literature, unpublished teaching aids, or citation-tracking (backward/forward reference searching) were included in the search process, which may have limited retrieval of studies not indexed in MEDLINE or Embase.

## 5 Conclusion

The Saint-Bonnet classification is a clinically logical and practical model for Doppler arterial waveform analysis. However, because all supporting evidence originates from a single, non-independent research group, its demonstrated utility should be regarded as promising rather than established; independent, multicentre validation is required before the classification can be considered for broader adoption. The capability of the model to create standardised descriptors, support functional assessment, and facilitate machine-learning automation demonstrates considerable value in vascular diagnostics, though prognostic utility remains to be established through longitudinal research.
