# Supplementary material for: Saint-Bonnet Doppler waveform classification system: a scoping review and evidence-based appraisal using AGREE II and AGREE-REX
Source: Front Cardiovasc Med. 2026 Oct 14;13:1870182. doi: 10.3389/fcvm.2026.1870182 (PMC13714507; doi:10.3389/fcvm.2026.1870182)
Supplement: Supplementary file 1 [file Datasheet1.pdf]

Supplementary Material

Saint-Bonnet Doppler Waveform Classification System: A Scoping Review and Evidence-Based Appraisal Using AGREE II and AGREE-REX

Alodayni H, Alodaini F, Davies AH, Normahani P

Supplementary Table S1. Full Database Search Strategy

Database: Embase Classic+Embase <1947 to 2025 November 04>, Ovid MEDLINE(R) ALL <1946 to November 04, 2025>

#	Search String	n
1	`((doppler or "hand-held doppler" or "continuous wave doppler" or "CW doppler") and (waveform* or triphasic or biphasic or monophasic) and classif* and ("lower limb" or "peripheral arter*")).mp.`	96
2	`("Saint Bonnet" or "Saint-Bonnet" or Spronk or Descotes).mp.`	61
3	`1 and 2`	16

Supplementary Table S2. Item-Level AGREE II and AGREE-REX Scores and Inter-Rater Agreement

Each item was scored independently by two reviewers (H.A. and F.A.) on a 7-point Likert scale (1 = strongly disagree, 7 = strongly agree). "Diff." indicates the absolute difference between reviewer scores for that item.

S2.1 AGREE II — Item-Level Scores

Domain	Item	Statement	H.A.	F.A.	Diff.
Domain 1: Scope and Purpose	Item 1	The overall objective(s) of the guideline is (are) specifically described.	6	6	0
Domain 1: Scope and Purpose	Item 2	The health question(s) covered by the guideline is (are) specifically described.	6	5	1
Domain 1: Scope and Purpose	Item 3	The population to whom the guideline is meant to apply is specifically described.	7	6	1
Domain 2: Stakeholder Involvement	Item 4	The guideline development group includes individuals from all relevant professional groups.	6	6	0
Domain 2: Stakeholder Involvement	Item 5	The views and preferences of the target population have been sought.	3	2	1
Domain 2: Stakeholder Involvement	Item 6	The target users of the guideline are clearly defined.	7	7	0
Domain 3: Rigour of Development	Item 7	Systematic methods were used to search for evidence.	3	2	1
Domain 3: Rigour of Development	Item 8	The criteria for selecting the evidence are clearly described.	2	3	1
Domain 3: Rigour of Development	Item 9	The strengths and limitations of the body of evidence are clearly described.	5	6	1
Domain 3: Rigour of Development	Item 10	The methods for formulating recommendations are clearly described.	5	4	1
Domain 3: Rigour of Development	Item 11	The health benefits, side effects, and risks have been considered.	4	5	1
Domain 3: Rigour of Development	Item 12	There is an explicit link between the recommendations and the supporting evidence.	4	3	1
Domain 3: Rigour of Development	Item 13	The guideline has been externally reviewed by experts prior to its publication.	4	4	0
Domain 3: Rigour of Development	Item 14	A procedure for updating the guideline is provided.	2	2	0

Domain	Item	Statement	H.A.	F.A.	Diff.
Domain 4: Clarity of Presentation	Item 15	The recommendations are specific and unambiguous.	7	7	0
Domain 4: Clarity of Presentation	Item 16	The different options for management are clearly presented.	7	7	0
Domain 4: Clarity of Presentation	Item 17	Key recommendations are easily identifiable.	6	7	1
Domain 5: Applicability	Item 18	The guideline describes facilitators and barriers to its application.	3	3	0
Domain 5: Applicability	Item 19	Advice and/or tools on how the recommendations can be put into practice are provided.	4	6	2
Domain 5: Applicability	Item 20	The potential resource implications of applying the recommendations have been considered.	3	4	1
Domain 5: Applicability	Item 21	Monitoring and/or auditing criteria are presented.	2	2	0
Domain 6: Editorial Independence	Item 22	The views of the funding body have not influenced the content of the guideline.	4	3	1
Domain 6: Editorial Independence	Item 23	Competing interests of guideline development group members have been recorded and addressed.	5	4	1

AGREE II Domain Scores

Domain	Scaled Domain Score (%)	Interpretation
Domain 1: Scope and Purpose	83%	High quality
Domain 2: Stakeholder Involvement	69%	Moderate quality
Domain 3: Rigour of Development	44%	Low quality
Domain 4: Clarity of Presentation	97%	High quality
Domain 5: Applicability	40%	Low quality
Domain 6: Editorial Independence	50%	Moderate quality

S2.2 AGREE-REX — Item-Level Scores

Domain	Item	Statement	H.A.	F.A.	Diff.
Domain 1: Clinical Applicability	Item 1	Relevance to vascular clinical practice is explicit.	6	6	0
Domain 1: Clinical Applicability	Item 2	Target population and setting (PAD, lower limb) are clearly supported.	7	6	1
Domain 1: Clinical Applicability	Item 3	Recommendations are consistent with current clinical pathways.	5	6	1
Domain 2: Values & Preferences	Item 1	Patient/clinician values are acknowledged where appropriate.	3	4	1
Domain 2: Values & Preferences	Item 2	Allows flexibility/adaptation to local contexts.	4	4	0
Domain 2: Values & Preferences	Item 3	Uncertainty or variability is transparently described.	5	6	1
Domain 3: Implementability	Item 1	Recommendations are actionable and specific.	6	5	1
Domain 3: Implementability	Item 2	Barriers/facilitators to implementation are discussed.	6	6	0
Domain 3: Implementability	Item 3	Practical tools or training/monitoring suggestions are provided.	5	5	0

AGREE-REX Domain Scores

Domain	Mean Domain Score (1–7)	Interpretation
Domain 1: Clinical Applicability	6.0	High quality
Domain 2: Values & Preferences	4.3	Moderate quality
Domain 3: Implementability	5.5	High quality

S2.3 Inter-Rater Agreement

Agreement between the two reviewers (H.A. and F.A.) was assessed using the intraclass correlation coefficient (ICC, two-way random-effects model, absolute agreement, single-rater), alongside percentage exact agreement, percentage agreement within one scale point, and the Pearson correlation coefficient.

Tool	Items (n)	Exact Agreement	Agreement Within 1 Point	Pearson r	ICC (2,1)
AGREE II	23	39.1%	95.7%	0.876 (p<0.001)	0.879
AGREE-REX	9	44.4%	100%	0.761 (p=0.017)	0.740

Interpretation (Koo & Li, 2016 ICC benchmarks): <0.50 poor; 0.50–0.75 moderate; 0.75–0.90 good; >0.90 excellent. Agreement was good-to-excellent for AGREE II (ICC = 0.879) and moderate-to-good for AGREE-REX (ICC = 0.740). Exact-match percentages appear comparatively low because the 7-point scale increases the number of possible score categories; the high within-one-point agreement (95.7% and 100%) and the ICC values indicate consistent scoring between reviewers.

Supplementary Table S3. Mapping of Original 13-Category to Simplified 8-Category Saint-Bonnet System

Original Category (13-type)	Simplified Category (8-type)	Morphological Description
**N**	**N**	Normal triphasic flow 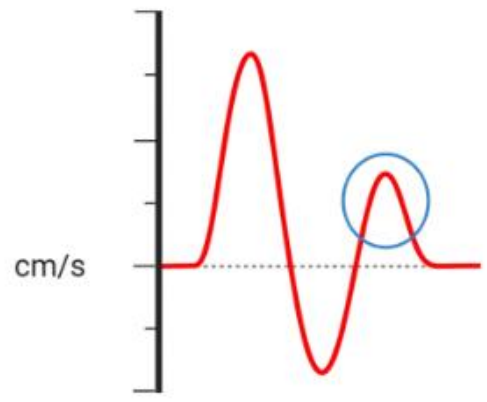
**A**	**A**	Loss of positive diastolic rebound (biphasic pattern) 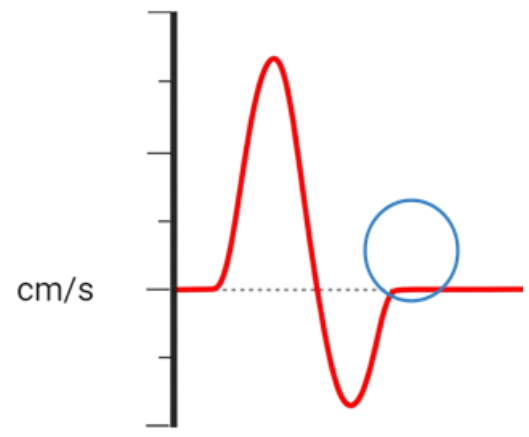
**B**	**B**	Symmetrical monophasic waveform with sharp systolic peak (loss of negative diastolic component) 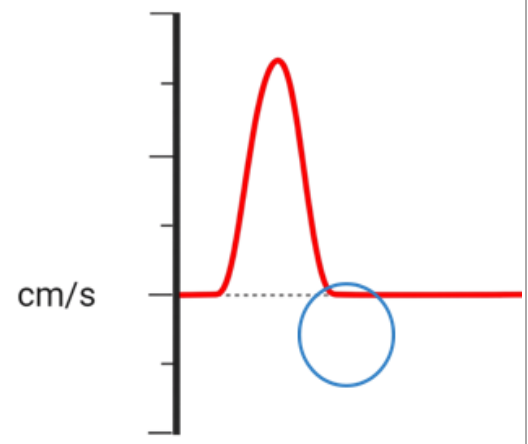
**C1**	**CD**	Asymmetrical monophasic waveform with ‘blunted’ systolic peak; descending branch does not occupy entire diastolic phase 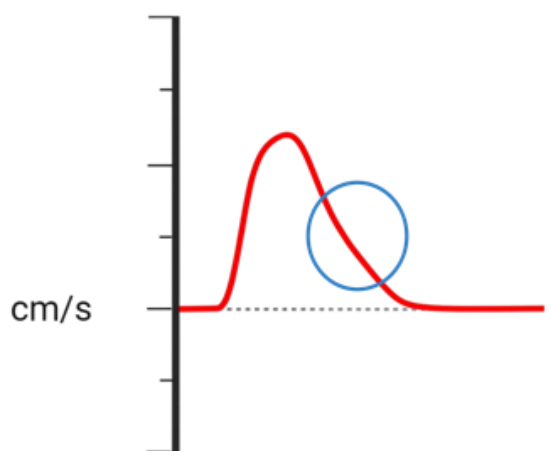

Original Category (13-type)	Simplified Category (8-type)	Morphological Description	
**C2**	**CD**	Asymmetrical monophasic waveform with ‘blunted’ systolic peak; descending phase occupies entire diastolic phase	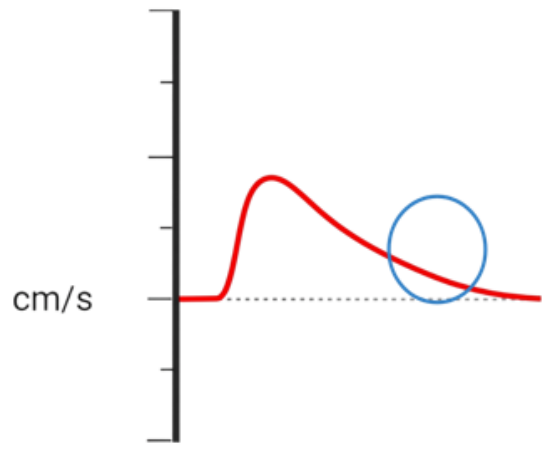
**D**	**CD**	Increased systolic rise time with ‘blunted’ systolic peak; severely attenuated low-amplitude monophasic flow	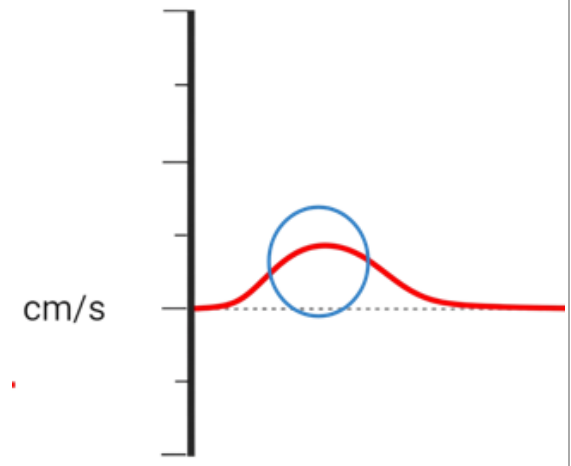
**E**	**E**	Loss of signal phase	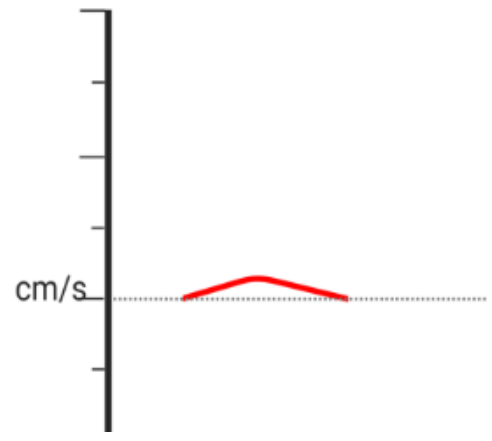
**O**	**O**	No waveform / absent flow	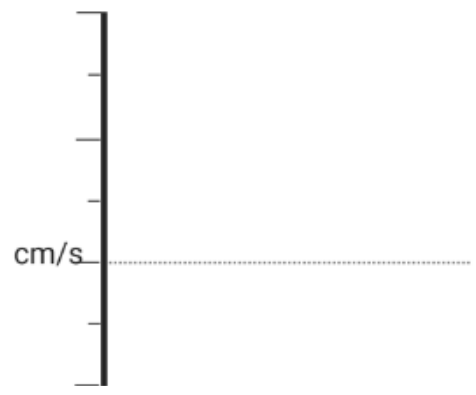
**FA**	**FA**	False aneurysm / turbulent flow pattern	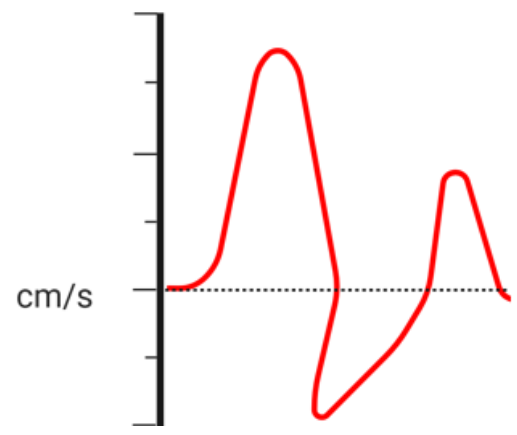
**U**	**U**	Atypical or unclassifiable pattern	NA
